# Meningitis: is vaccine introduction enough?

**DOI:** 10.1016/j.lansea.2026.100818

**Published:** 2026-07-10

**Authors:** The Lancet Regional Health – Southeast Asia

Meningitis, an infectious disease involving inflammation of the meninges, continues to cause severe morbidity and mortality globally. The WHO South-East Asia region bears one of the highest burdens, contributing to 27% of global incidence and 19% of mortality. Globally and regionally, bacterial meningitis has declined, largely due to the scale-up of the *Haemophilus influenzae* type b (Hib) vaccine and pneumococcal conjugate vaccine (PCV). However, 2023 Global Burden of Disease analysis on meningitis has highlighted that the decline in meningitis incidence and mortality has slowed since 2015, with viral aetiologies, particularly non-polio enteroviruses, emerging as the leading global cause.

Considering the evolving landscape of meningitis causing pathogens, there is a need for robust data generation on aetiological agents, which is essential not only for accurate diagnosis and effective patient management, but also for robust pathogen surveillance to guide future vaccine development based on serotype circulation. Currently, there are no established viral meningitis surveillance networks across the region. This gap is further complicated by the self-limiting nature of the disease and the endemic presence of Japanese encephalitis, which makes accurate diagnosis for viral meningitis even more challenging. Data from the WHO-coordinated Global Invasive Bacterial Vaccine-Preventable Diseases (IB-VPD) Surveillance Network, including countries from southeast Asia, has highlighted that post-PCV rollout, more than half of pneumococcal meningitis cases were caused by non-vaccine serotypes of *Streptococcus pneumoniae*.

In an era of foreign aid cuts, the responsibility is with countries to devise effective sustainable strategies to strengthen surveillance. Lessons can be drawn from other low-income and middle-income settings such as Africa, where high burden of meningococcal meningitis led to the establishment of a surveillance consortium, MenAfrinet, following the introduction of MenAfriVac vaccine. Although the rapid response to control meningitis outbreak in the African region might have triggered greater health system response, it offers a valuable blueprint for replication in similar settings. Meningitis surveillance could be integrated into existing vaccine-preventable disease surveillance, with strengthened mandatory notifications from all health-care providers, supported by a robust laboratory network capable of offering testing facility for multiple pathogens and serotypes. A parallel can be seen in countries expanding COVID-19 surveillance to cover other viral infections like influenza.

A hospital-based sentinel surveillance network was initiated in India in 2019, and in this issue of *The Lancet Regional Health – Southeast Asia*, Purushothaman and colleagues present preliminary findings on pneumococcal meningitis among hospitalised children. They demonstrate a decline in the proportion of pneumococcal meningitis and a decrease in cases attributable to PCV-covered serotypes in the post-PCV period compared with the pre-PCV period. While these data are valuable, there is an urgent need to strengthen such evidence, by increasing access to diagnostic services and enhancing representation through enrolling additional sites. Implementation of surveillance network for meningitis has faced several challenges in India, including variation in case definitions, difficulties in specimen collection, and limited access to specialist care. A persistent constraint remains the establishment of a reliable system for cerebrospinal fluid (CSF) sample collection, storage, transport, and testing.

In India and other countries of the region, CSF-based diagnosis for meningitis is available in selected tertiary care centres and is limited by its coverage of pathogen testing. Several tools have been under development, such as multiplex real-time PCR assays (simultaneous testing of multiple targets), which preserve samples, and reduce time and consumable costs. They detect multiple pathogens, including Hib, meningococci, and pneumococci in one reaction and can be used to test CSF directly without the need for DNA extraction. However, these have been validated in limited samples and their performance in varied clinical settings, populations, and geographies remains unknown. Lack of accurate and timely diagnosis could negatively impact outcomes and survival; it is therefore equally important to develop and invest in low-cost, robust, easy to use point of care diagnostic tools.

Antimicrobial resistance increasingly poses a key challenge in the first line of treatment for meningitis. A robust surveillance network could track emerging resistance patterns and provide the evidence base to guide effective management strategies. There is currently no existing data on long-term sequelae following meningitis. The study by Purushothaman and colleagues demonstrates that 93.1% of children in their study recovered, however it is important to generate follow-up data, as emerging research shows increased risk of neurodevelopmental impairment among survivors.

The current strategies for tackling meningitis in the region rely heavily on available vaccines; given the evidence on multiple and evolving aetiological agents, uneven vaccine coverage, and absence of robust data, there is an urgent need for multipronged approach. As outbreaks of vaccine-preventable diseases continue to rise, and considering the threat of AMR, it is high time countries invest in locally rooted, sustainable strategies to reach the ambitious target set by WHO in defeating meningitis. A starting point could be devising comprehensive national meningitis plans, leveraging existing infrastructure through cross-cutting synergistic solutions catering to surveillance of multiple vaccine-preventable diseases.

